# The Influence of Thermal Treatments on Anchor Effect in NMT Products

**DOI:** 10.3390/polym14091652

**Published:** 2022-04-20

**Authors:** Huazheng Li, Linling Li, Ye Sha, Yuyuan Lu, Chao Teng, Dongshan Zhou, Wei Chen, Gi Xue

**Affiliations:** 1 Department of Polymer Science and Engineering, State Key Laboratory of Coordination Chemistry, Key Laboratory of High Performance Polymer Materials and Technology of Ministry of Education, Nanjing University, Nanjing 210023, China; mg1924041@smail.edu.cn (H.L.); shaye@njfu.edu.cn (Y.S.); dzhou@nju.edu.cn (D.Z.); xuegi@nju.edu.cn (G.X.); 2 Institute of Critical Materials for Integrated Circuit, Shenzhen Polytechnic, Shenzhen 518055, China; LinlingLi@szpt.edu.cn (L.L.); tengchao@szpt.edu.cn (C.T.); 3 Department of Chemistry and Material Science, Nanjing Forestry University, Nanjing 210037, China; 4 State Key Laboratory of Polymer Physics and Chemistry, Changchun Institute of Applied Chemistry, Chinese Academy of Sciences, Changchun 130022, China; yylu@ciac.ac.cn

**Keywords:** anchor effect, nanomolding technology, interfacial fluorescence resonance energy transfer, interfacial bonding strength

## Abstract

The anchor effect in nanomolding technology (NMT) refers to the effect that polymer nanorods in nanopores on metal surfaces act as anchors to firmly bond the outside polymer components onto the metal surface. In this work, the influences of thermal treatments on the anchor effect are studied at microscopic level from the perspective of interfacial interaction by a model system (poly(*n*-butyl methacrylate) (PBMA) and alumina nanopore composite). The differential scanning calorimeter and fluorescence results indicate that the formation of a dense polymer layer in close contact with the pore walls after proper thermal treatments is the key for a strong interfacial interaction. Such polymer layers were formed in NMT products composed of PBMA and aluminum after slow cooling or annealing, with an up to eighteen-fold improvement of the interfacial bonding strength. The polymer chains near the nanopore walls eliminate the thermal stress induced by the mismatch of thermal expansion coefficients through relaxation over time and remain in close proximity with the pore walls during the cooling process of nanomolding. The above dynamic behaviors of the polymer chains ensure the formation of stable interfacial interaction, and then lead to the formation of the anchor effect.

## 1. Introduction

Influenced by the urgent requirements for improving the interfacial properties of organic/inorganic integrated materials, considerable attention has been paid to manufacturing technologies that enable dissimilar materials to be firmly joined together without the use of chemical adhesives [1,2,3,4]. Among them, nanomolding technology (NMT) is a novel dissimilar joining technology that can produce polymer/metal integrated materials through an insert injection molding [5,6,7,8,9,10]. In a typical nanomolding process, the metallic component is etched to produce nanopores on the surface. The resulting surface-modified metallic component is inserted into a mold and then polymer melt is directly injected onto the metal surface and fills nanopores under pressure. After cooling and demolding, the polymer/metal integrated material, namely “NMT product”, is obtained [11,12]. Taking merit from its thin interfacial thickness and ultrahigh bonding strength, the NMT product is a promising material to satisfy the increasing demands for lightweight vehicles and highly integrated electronics [13,14]. The polymer fillers located inside the interfacial nanopores act as anchors to bond bulk polymer components with metal substrate, resulting in ultrahigh bonding strength attributed to the so called “anchor effect” [15,16]. However, during the production and life cycle of most sorts of general plastics, an inefficient anchor effect often occurs in these NMT products, resulting in material failure. Up to now only four polymers (i.e., polyphenylene sulfide, polybutylene terephthalate, polyamide 6 and polyamide 66) [14,17] have been reported to be successfully used as polymer components in NMT products with stable bonding strength.

A mechanistic understanding of the failure mechanism for the anchor effect at the micro level is a prerequisite to predicting the interfacial performance of NMT products. The macroscopic interface between the polymer and metal component of an NMT product consists of metal oxide nanopores and polymer fillers located in the etched pores. Apparently, at the microscopic level, the strength of the anchor effect is determined by the interfacial interaction between the polymer nanorods and the inorganic etched pore walls. Such interfacial interaction can be significantly affected by the proximity of polymer fillers and the pore walls. The proximity may be altered due to the mismatch of coefficients of thermal expansion (CTEs), since the CTE of the polymer is usually 1 to 2 orders of magnitude higher than that of the inorganic substrate [18,19]. During the cooling process, if the shrinkage of the polymer melt is isotropic, the mismatch of CTEs would result in a gap between the polymer fillers and the pore walls. For instance, as reported by Teng et al. [20] the gap between polystyrene (PS) nanorods within the porous anodic alumina oxide (AAO) nanopores (300 nm diameter) after cooling from 180 °C to 25 °C, is estimated to be 6 nm, assuming that the PS nanorods shrink isotropically. At this scale, the interfacial interaction such as the hydrogen bonding and van der Waals force, between the polymer nanorods and the inorganic pore walls can be neglected. It is worth noting that the proximity between polymer nanorods and pore walls can be significantly affected by the CTE mismatch-induced thermal stress generated during the cooling process. The thermal stress also leads to varying dynamic behaviors of the polymer chains near the pore walls. Since the nonequilibrium glassy state of polymer nanorods depends on the path in which the glass is formed, control over the dynamic behaviors of polymer chains during the cooling process could regulate the shrinkage mode of polymer nanorods. Based on the above analysis, a physical image on the failure of anchor effect can be hypothesized as follows: during the injection process of nanomolding, the nanopores are filled with polymer melt which form an adsorbed layer in close contact with the pore walls [21]. In other words, polymer chains near pore walls are in close proximity with the pore walls. During the subsequent cooling process, the volume shrinkage of the polymer nanorods brings additional thermal stress imposed by the wall. Since the interfacial thermal stress cannot be dissipated in a timely fashion, the polymer chains near the pore walls are subjected to high stress along the radial direction of the nanopores which will completely/partly peel off from the wall. Then the interfacial interaction between the polymer nanorods and pore walls most likely decreases, resulting in the failure of the anchor effect. Therefore, the key to avoid the failure of the anchor effect depends on the dissipation of the thermal stress accumulated at the interface during the cooling process of the NMT products.

Proper thermal treatments are effective ways to eliminate thermal stress. Teng et al. [20] investigated the influence of thermal stress on the glass state of PS nanorods in AAO nanopores, various glass states of polymer nanorods in nanopores were obtained by controlling the thermal treatment process. For example, Li et al. [22,23] and Zhang et al. [24] reported the emergence of two glass transition temperatures (*T_g,low_* and *T_g,high_*) of poly(methyl methacrylate) (PMMA) nanorods confined in AAO nanopores after an ultraslow cooling process. A heterogeneous core-shell structure of PMMA nanorods has been proposed to explain the two *T_g_*s phenomenon. Sha et al. [25] demonstrated that the poly(*n*-butyl methacrylate) (PBMA) oligomer forms an adsorbed layer with closer interchain proximity relative to the bulk via high temperature infiltrating and slow cooling. Although these heterogeneous glassy states of polymer nanorods confined in hard nanopores have been regarded as the result of the combination of interfacial interaction and the thermal treatment process, the experimental evidence in previous work focused on the structure characteristics of polymer nanorods. There is no direct experiment evidence that shows the proximity between the polymer chains and nanopore walls can be altered by thermal treatment due to the lack of a suitable characterization method.

In this work, we utilized interfacial fluorescence resonance energy transfer (i-FRET) to detect the proximity between the polymer chains and pore walls by labeling donor and acceptor fluorophores on the polymer chains and the walls of nanopores, respectively. PBMA confined in AAO nanopores was used as a model system to investigate the influences of thermal treatments on the proximity between the polymer chains and pore walls. Due to the positive correlation between the proximity and interfacial interaction, the influences of thermal treatments on the anchor effect of NMT products composed of PBMA and aluminum are also discussed.

## 2. Materials and Methods

### 2.1. Preparation of PBMA-Cz@AAO-an Samples for i-FRET Measurements

According to reference [26], PBMA samples labeled with carbazolyl chromophores (PBMA-Cz) were prepared using an atom transfer radical polymerization (ATRP) method with random labeling of trace amounts of fluorescent monomers. The PBMA-Cz samples had a weight-average molecular weight of 73,900 Da with a polydispersity index of 1.38 according to measurement on PL-GPC 120 (Polymer Laboratories Inc., Long Beach, CA, USA). Ultraviolet absorption spectrum was measured using a PerkinElmer Lambda 35 UV−vis spectrophotometer (PerkinElmer, Waltham, MA, USA). The carbazolyl-labeled monomer unit concentration in mole percent was 0.435%.

**Figure 1:**
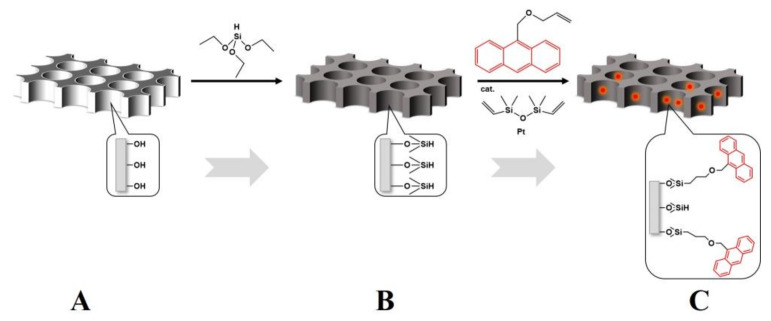
Labeling fluorophores on nanopore walls of AAO. (**A**) Hydrogen peroxide treatment results in a large amount of hydroxyl groups on the pore walls of AAO. (**B**) Hydroxyl groups coupled with triethoxysilane to prepare reactive hydrogen sites. (**C**) Anthryl-labeled pore walls. The red solid luminous dots represent the anthryl chromophores.

As shown in Figure 1, the AAO membranes purchased from Hefei PUYUAN Nano Ltd. (PUYUAN, Hefei, Anhui, China) with an average pore size of 200 nm and thickness of 80 μm were labeled with anthryl chromophore through 3 steps of surface modifications. The AAO membranes were rinsed with 70% hydrogen peroxide solution at 50 °C for 30 min to hydroxylate the nanopore walls in step 1. Then the hydroxylated AAO membranes were reacted with triethoxysilane in dry tetrahydrofuran solution for 3 days to give reactive hydrogen sites in step 2. Subsequently, the reactive AAO membranes were put in dry tetrahydrofuran solution to react with vinyl anthritol through a hydrosilylation reaction at 30 °C for 3 days using platinum catalyst to label anthryl chromophores on the nanopore walls of AAO membranes. All above reactions were carried out in nitrogen atmosphere. The anthryl chromophores labeled AAO were denoted as AAO-An. The PBMA-Cz powder was dissolved in redistilled toluene at a concentration of 10 wt% and stirred vigorously overnight. A 60 μm precursor film was cast onto a smooth glass sheet and then placed in a clean fume hood for 3 days to allow for solvent evaporation. Subsequently, it was dried under vacuum for 12 h at 80 °C. Then, the dried PBMA-Cz film was placed on top of the AAO-An membrane and heated at 105 °C under high vacuum for 12 h to obtain fully wetted nanorods. Then, the samples were cooled at different rates (fast cooling was achieved by rapidly transferring the hot samples into liquid nitrogen, and the cooling rate was estimated to be 6000 °C min; slow cooling was normally cooled directly in the oven equipment with a cooling rate of ~1 °C/min). The residual PBMA on the top of AAO template was carefully scraped with a surgical blade. Such PBMA-Cz filled AAO-An samples were denoted as PBMA-Cz@AAO-An. All the samples were stored at –25 °C after cooling.

### 2.2. Preparation of PBMA@AAO Samples for DSC Measurements

PBMA purchased from Shanghai Macklin Biochemical Co., Ltd (Macklin, Shanghai, China) with a weight-average molecular weight of 66,000 Da) and the AAO membranes with average pore sizes of 200 nm and thickness of 80 μm were used to prepare PBMA filled AAO samples, which were denoted as PBMA@AAO. The preparation method is the same as that of PBMA-Cz@AAO-An.

### 2.3. Preparation of NMT Products

**Figure 2:**
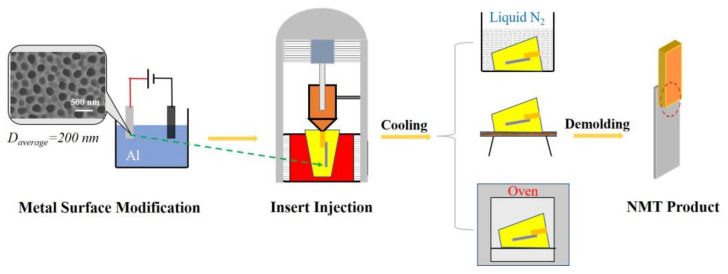
The main steps of nanomolding and corresponding NMT product. The left illustration is the SEM image of the aluminum sheet after surface modification, and more details of surface morphology can be found in Figure S1.

According to reference [27], NMT products were prepared through a series of steps. As shown in Figure 2, an aluminum sheet (Al5052) with dimensions of 50 mm × 15 mm × 1 mm was processed in 0.2 M H_3_PO_4_ solution as an anode by two-step anodization at −2 °C to generate alumina nanopores on the surface. Before anodization, the sheet was electrochemically polished with solution of 12 wt% CrO_3_ and 80 wt% H_3_PO_4_ at a current density of 120 mA/cm^2^ to remove the natural oxide layer. In the first anodization, the sheet’s surface was oxidized and formed a thin oxide layer, then the oxide layer was removed by immersion in an acidic solution. In the second anodization, the formation and dissolution of the oxide layer occurred simultaneously at the top and bottom of the roughened layer with a voltage of 165 V, forming approximately regular pores on the surface. Then, the aluminum sheet was immersed in a solution of 5 wt% H_3_PO_4_ to increase the pore diameter. SEM results show that the average diameter of the prepared nanopores was about 200 nm, as shown in Figure 2. The surface-modified aluminum sheet was then inserted into a mold and polymer melt was injected onto the surface of the sheet by the injection molding machine (WZS05, Shanghai XINSHUO Precision Machinery Co., Ltd., Shanghai, China). After cooling and demolding, the NMT product with a bond area of 50 mm^2^ for the evaluation of the bonding strength was manufactured.

Three sorts of polymers, i.e., poly(phenylene sulfide) (PPS, 1135ML, Japan Poly Plastics Co., Ltd, Tokyo, Japan), poly(methyl methacrylate) (PMMA, injection grade with a weight-average molecular weight of 47,000 Da Shanghai Macklin Biochemical Co., Ltd, Shanghai, China), and poly(*n*-butyl methacrylate) (PBMA, injection grade with a weight-average molecular weight of 66,000 Da Shanghai Macklin Biochemical Co., Ltd., Shanghai, China) were used to manufacture corresponding NMT products. Corresponding melt temperatures of injection molding were 315 °C, 240 °C, 200 °C, respectively, and the mold temperatures were 140 °C, 140 °C, 80 °C, respectively. All NMT products were denoted as polymer/Al. After injection, the PBMA/Al was cooled to −25 °C, the PPS/Al and PMMA/Al were cooled to room temperature. NMT products were cooled with different cooling rates (fast cooling was achieved by rapidly transferring NMT products into liquid nitrogen, the cooling rate was estimated to be 1000 °C/min; ambient cooling was achieved by putting the NMT product directly in atmosphere with an estimated cooling rate of ~10 °C/min; slow cooling was achieved in the oven with a cooling rate of ~1 °C/min). The manufactured NMT products were used as test specimens for the evaluation of the bonding strength according to ISO19095 standard.

### 2.4. Characterization

i-FRET measurements. After the specified thermal treatment, reflectance fluorescence spectra were collected with a PTI QM40 fluorometer (Photon Technology International Inc., Birmingham, NJ, USA) at an excitation wavelength of 294 nm. The band-pass excitation and emission slits were both 0.25 μm. The fluorescence emission intensity was collected from 300 nm to 500 nm.

Thermal analysis. For differential scanning calorimeter (DSC, Mettler Toledo DSC1, Zurich, Switzerland) measurements, the weight of the filled AAO sample was approximately 20 mg, and the heating rate was 10 °C/min. The temperature was calibrated with indium and zinc standards before measurements. For thermomechanical analysis (TMA Q400, TA Instruments, New Castle, DE, USA) test, PBMA (approximately 50 mg) were heated from −20 °C to 45 °C at 3 °C/min.

SEM measurements. A Hitachi S−4800 scanning electron microscope (Hitachi, Tokyo, Japan) with an accelerating voltage of 5 kV was used to investigate the nanostructure morphologies. All samples were coated with several nanometers of Au before performing SEM measurements.

Bonding strength measurements. The bonding strength of NMT products were measured by AG-X plus electronic universal testing machine (Japan SHIMADZU Co., Ltd., Kyoto, Japan) with special clamps, and the tensile speed was 2 mm/min. All tests were carried out at room temperature except for PBMA/Al, which was tested at −25 °C.

## 3. Results and Discussion

The thermal treatments’ influence on the interfacial interaction of polymer confined in nanopores were studied by using PBMA@AAO as the model material.

### 3.1. Theoretical Calculation of the Interfacial Interaction

**Figure 3:**
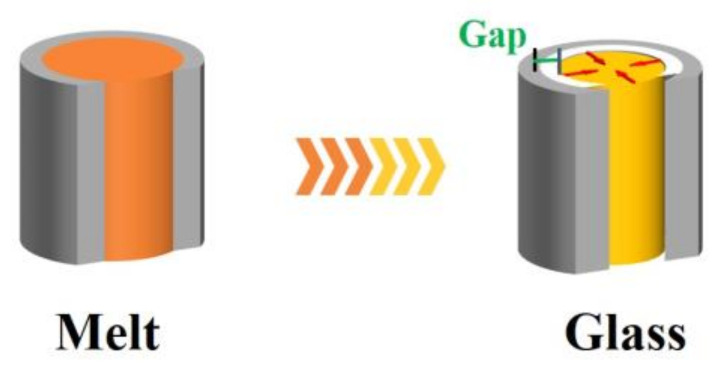
Isotropic shrinkage of PBMA nanorods from melt to glass. The orange and yellow cylinders represent the polymer melt, and the polymer glass, while the grey hollow cylinder represents the alumina nanopore.

When polymer melt is cooled into glass, volume shrinkage happens when the temperature decreases. If the interfacial interaction between the polymer nanorods and pore walls is not taken into account, the interfacial proximity will be reduced due to the difference in shrinkage between the inorganic substrate and the polymer nanorods. For instance, the density change of bulk PBMA during cooling is estimated from the empirical equation below: [28,29]

(1) $$\rho =1.0695-5.82\times 10^{-4}t-0.98\times 10^{-6}t^{2}+0.241\times 10^{-8}t^{3}\left(T_{g}<t<200\;℃\right)$$

(2) $$\rho =1.063-4.01\times 10^{-4}t\;(-30\;℃<t<T_{g})\;$$

where t represents the centigrade temperature. This leads to a about 13% change in volume over the whole experimental temperature range (from 200 °C to −25 °C), while for the alumina, the dimension change is less than 0.5%. If the initial diameter of the PBMA rods is 200 nm, then the gap between the PBMA nanorods and pore walls after cooling to −25 °C is estimated to be about 4.5 nm (assuming that the PBMA nanorods shrink isotropically, Figure 3). At this scale, the interaction (such as the hydrogen bonding and van der Waals force) between the PBMA chains and pore walls could be neglected.

**Figure 4:**
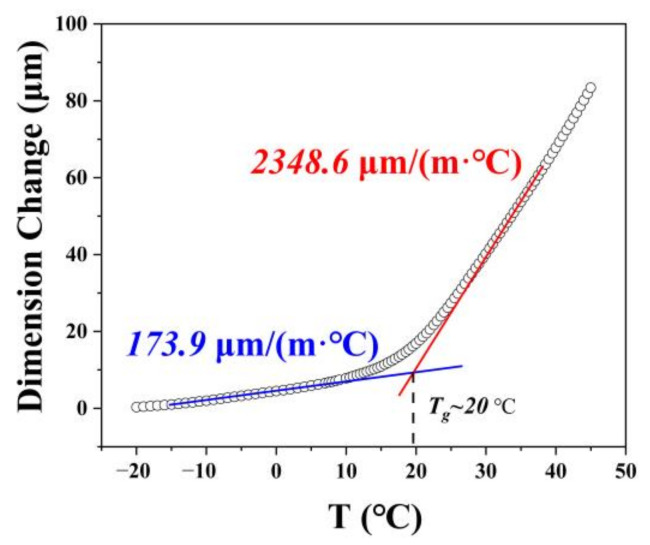
The relationship between dimension change and temperature of PBMA, and the CTEs of PBMA in glass state and high elastic state are calculated, respectively.

Actually, when the polymer melt fills in the nanopores, an absorbed layer with a thickness of several nanometers is formed, making the polymer chains in close proximity to the pore walls to produce strong interfacial interactions [21]. As the melt gradually cools into glass, the coefficient of thermal expansion (CTE) changes drastically near the T_g_, as shown in Figure 4. For polymer/inorganic substrate integrated material, the CTE of the polymer is usually orders of magnitude higher than that of the inorganic material. In our case, the CTEs of PBMA (173.9 μm/(m·°C), 2348.6 μm/(m·°C), below and above T_g_, respectively) were dozens or even hundreds of times more than that of alumina (7.7 μm/(m·°C)) [30] in the corresponding temperature range. During the cooling process of the polymer from melt to glass, the significant mismatch of the CTEs is very likely to lead to the accumulation of thermal stress at the interface.

In addition, the polymer nanorods were surrounded by hard pore walls, which can be roughly regarded as an isochoric environment. In such a constraint environment, the thermal stress of polymer confined in nanopores in the cooling process can be calculated according to the formula proposed by Teng et al. [20] as follows:

(3) $$\sigma \left(t\right)=\alpha Q\int_{0}^{t}G\left(t-\;t\;\text{′},T\left(\;t\;\text{′}\right)\right)d\;t\;\text{′}$$

(4) $$G\left(t\right)=\frac{8}{\pi^{2}}G_{e}\exp\left(-t/\tau_{d}\right)$$

with $G_{e}=\rho \mathrm{RT}/M_{e}$, where α is the CTE, Q is the cooling rate, $\tau_{d}$ is the terminal relaxation time, $G_{e}$ is the plateau modulus, ρ is the density of PBMA, R is the molar gas constant, and $M_{e}$ is the entanglement molecular weight of PBMA (30,000 g/mol) [31]. The $\tau_{d}$ obey the Vogel–Fulcher–Tamman (VFT) law:

(5) $$\tau \left(T\right)=\tau_{0}\exp\left({\mathrm{BT}}_{0}/(T-T_{0}\right))$$

where $T_{0}$ is the Vogel temperature which defines the temperature with infinite relaxation time, B is a constant depending on materials, and $\tau_{0}$ is the pre-exponential factor. For PBMA, the detailed parameters are $\tau_{0}=10^{-3.3}\;s$, B=1.41, $T_{0}=255\;K$ [32] As calculated for PBMA cooled from 200 °C to −25 °C at a fast cooling rate, the thermal stress was as great as ~90 MPa, which would overcome the yield stress of PBMA (~23 MPa) [33], and peel the chains away from the wall, leading to the decrease of interfacial interaction. This would reduce the proximity between the PBMA chains near the interface and the alumina pore walls. On the other hand, once the cooling rate is sufficiently slow, the CTE-induced thermal stress would be relaxed over time and the PBMA can keep in close contact with the pore walls during the cooling process.

### 3.2. The Thermal Treatments Dependence on the Interfacial Proximity

The interfacial interaction between the polymer nanorods and pore walls depends on the proximity between the polymer chains near the pore walls and the pore walls. In the process of thermal treatment, ensuring close proximity is the key to obtaining strong interfacial interaction. However, the distance between the polymer chains near the pore walls and the pore walls is generally several nanometers, which challenges the sensitivity of the traditional characterization method.

Fluorescence resonance energy transfer (FRET) spectroscopy provides a sensitive strategy to detect distance changes at the nanoscale [34,35,36,37,38]. FRET is a non-radioactive energy transfer phenomenon that occurs between donor and acceptor fluorophores through long-term dipole–dipole interaction. The relationship between energy transfer efficiency (E) and the distance between donor and acceptor fluorophores (r) is described by the Förster formula: [39]

(6) $$E={\left(1+{\left(r/R_{0}\right)}^{6}\right)}^{-1}$$

where $R_{0}$ is the Förster critical radius, which is only related to the selected fluorophores. For most fluorophores, the value of $R_{0}$ is between 1 nm and 4 nm, so FRET can provide information for interfluorophore distance in the range of 0.5−10 nm, [34] resulting in the description of FRET as a “spectroscopic ruler” at the nanoscale [40,41]. Since E is positively correlated with the ratio of the acceptor emission peak intensity to the donor emission peak intensity, this ratio is often used to qualitatively analyze the distance change experimentally [36,42]. Obviously, the larger intensity ratio correlates with a closer donor–acceptor distance [39]. Herein, we use i-FRET method to detect the interfacial proximity by labeling the donor (carbazolyl) and acceptor (anthryl) on PBMA chains and AAO pore walls, respectively.

**Figure 5:**
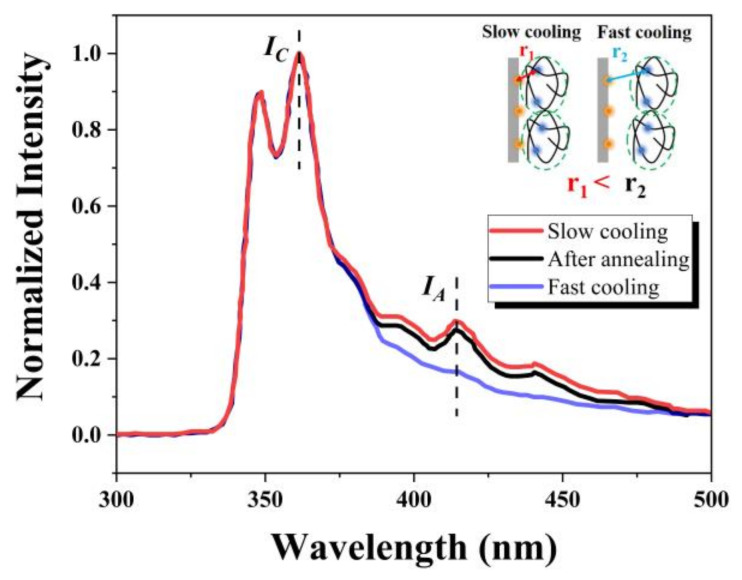
Fluorescence spectra of PBMA-Cz@AAO-An after different thermal treatments. The interfacial proximities after slow cooling, fast cooling and annealing are illustrated therein.

As shown in Figure 5, obvious differences exist in the i-FRET signals of PBMA-Cz@AAO-An after different cooling rates. The emission peak at 362 nm (marked as I_C_) is the characteristic peak of the donor carbazolyl, while that at 414 nm (marked as I_A_) is the characteristic peak of the acceptor anthryl. Here we normalized I_C_ to directly compare I_A_. The I_A_ of PBMA-Cz@AAO-An after slow cooling was much stronger than that of the sample after fast cooling, indicating that the interfacial proximity of the slow cooling sample was closer than that of the fast cooling sample. If the fast cooling PBMA-Cz@AAO-An was annealed at 80 °C for 2 h and then slowly cooled to −25 °C, the I_A_ of PBMA-Cz@AAO-An after annealing was significantly enhanced and reached the same level as the slow cooling sample, indicating that the interfacial proximity of the fast cooling sample could be enhanced by annealing. These results demonstrate that thermal treatments such as slow cooling and annealing are efficient ways to obtain closer interfacial proximity between the polymer chains and pore walls. The reason for the influence of thermal treatments on the interfacial proximity depends on the alteration of accumulated thermal stress at the interface induced by the CTE mismatch during the cooling process. Since the thermal stress is cooling rate dependent, slow cooling is beneficial to the timely dissipation of thermal stress. The thermal stress accumulated during fast cooling can be gradually dissipated by annealing through the accelerated motion of the polymer segments at a temperature above T_g_. It can be expected that once the cooling rate is sufficiently slow, the CTE-induced thermal stress may relax over time during the cooling and the polymer chains are kept in contact with the pore walls during the cooling process.

### 3.3. The Thermal Treatments Dependence on the Glassy States

In order to further explore the changes of the glassy state of polymer nanorods after different thermal treatments, we performed DSC tests on the three PBMA@AAO samples after the same thermal treatment as performed on the corresponding PBMA-Cz@AAO-An samples.

**Figure 6:**
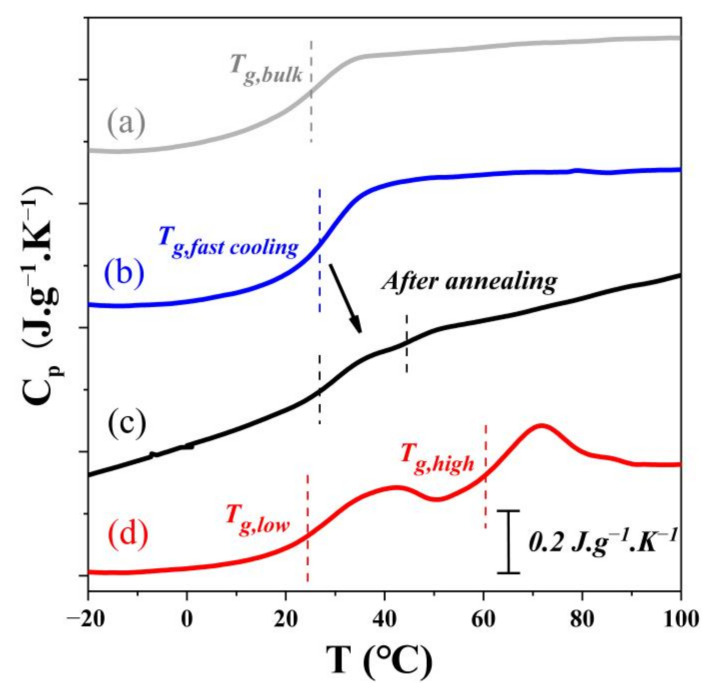
The DSC curves for the PBMA@AAO after different thermal treatments. (**a**) The DSC curve of bulk PBMA; the DSC curves of the PBMA@AAO (**b**) after fast cooling; (**c**) after annealing; (**d**) after slow cooling.

Figure 6 shows the DSC heating thermograms of these samples. Two separate T_g_s were obtained after a slow cooling, and the lower T_g_ (denoted as T_g,low_) at 25 °C was similar to that of the bulk T_g_ while the higher T_g_ (denoted as T_g,high_) was approximately 60 °C. Deviations between the two T_g_s could be as large as 35 °C, which indicates significant heterogeneity of PBMA nanorods confined in the nanopores. A core-shell structure can be used to explain the two T_g_s phenomenon of nanorods after slow cooling. As reported before, the glass transition behaviors of polymer chains confined in nanopores can be significantly affected by the cooling rate [22,23]. When slow cooling is performed, the thermal stress can be relaxed sufficiently, and the adsorbed layer formed in melt keeps in contact with the surface of the pore walls instead of being peeled off from the pore wall as observed during fast cooling. The influence of interfacial interaction on polymer chain dynamics could propagate a much longer distance by long-range effects such as topological entanglement [43]. After slow cooling, the PBMA filled into the pores forms a dense interfacial layer with poor mobility and high T_g_ [44,45,46]. In another words, in a heterogeneous glass of PBMA nanorods, the polymer chains near the pore walls still keep close proximity to the pore walls, leading to strong interfacial interactions.

In addition, two T_g_s can also be found in the DSC of annealed sample. Li et al. claimed that the interface behaviors were reversible, only depending on the thermal history [22]. This means that the homogeneous glass can transform into heterogeneous glass after annealing to restore the close proximity between the polymer chains near to the pore walls and the pore walls. We noticed that the thermal stress cannot be completely relaxed during this annealing experiment, as reflected in the close value of two T_g_s, indicating the interfacial layer has been generated, but still in progress under such annealing conditions, which is in reasonable agreement with the i-FRET results.

### 3.4. The Thermal Treatments Dependence on the Interfacial Interaction

**Figure 7:**
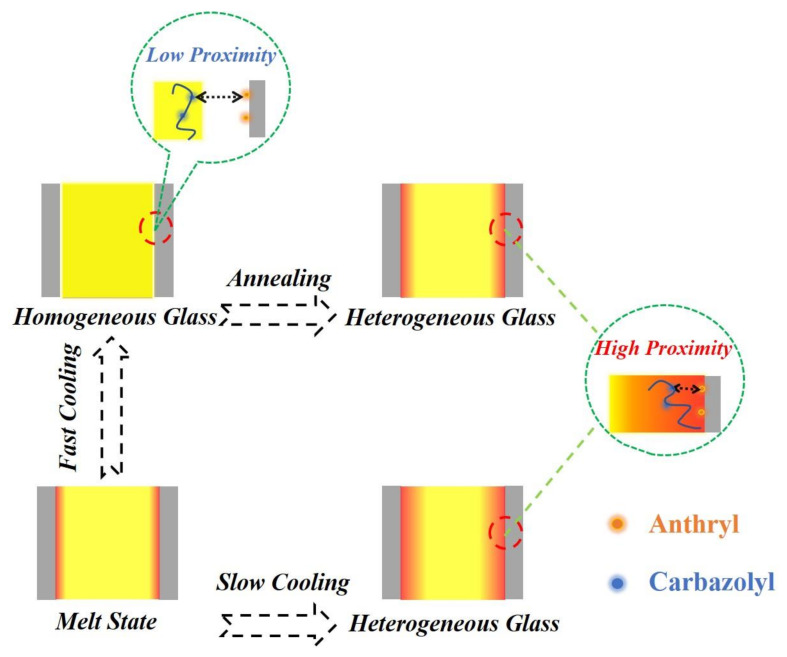
The evolution of nanostructure of polymer nanorods confined in nanopores during the cooling process from melt to glass. The solid luminous dots represent the fluorophores, with red dots as donors and blue dots as acceptors. The red area represents the absorbed layer, and the yellow area represents the bulk-like layer.

Based on the above results and analysis, we can clearly describe the effects of different thermal treatments on the interfacial interaction between confined polymers and nanopore walls. As shown in Figure 7, when the polymer melt is filled in the nanopores, an adsorbed layer will be formed at the interface. The cooling rate is an important factor to regulate the interfacial interaction during the cooling process. During fast cooling, the thermal stress caused by the mismatch of CTEs cannot be relaxed sufficiently. If the accumulated thermal stress exceeds the critical value of the yielding stress of the polymer filler, the adsorbed layer will be peeled away from the pore walls, resulting in the decrease of interfacial interaction. After fast cooling, the homogeneous glass of nanorods confined in nanopores was formed. In contrast, the thermal stress can be relaxed sufficiently during slow cooling, and the adsorbed layer will always maintain close proximity to the pore walls. With the interfacial interaction, the polymer nanorods finally form a heterogeneous glass. In addition, annealing can relax the accumulated thermal stress of homogeneous glass and reform a heterogeneous glass. Therefore, in order to obtain a strong interfacial interaction between the polymer nanorods and the pore walls, it is necessary to ensure the polymer melt forms a heterogeneous glass with a dense interfacial layer in close contact with the pore walls during the cooling process. Thermal treatments such as slow cooling and annealing can efficiently promote the formation of heterogeneous glass during the cooling process.

### 3.5. The Thermal Treatment Influence on the Bonding Strength of NMT Products

The dynamic behaviors of polymer nanorods can be regulated during the cooling process through proper thermal treatments, to ensure close proximity between the polymer chains near to the pore walls and the pore walls. This rule can be applied to nanomolding technology to improve the anchor effect of NMT products. In order to illustrate the influence of cooling rate on the macro performance of NMT products, we studied the relationship between the bonding strength of PBMA/Al and the cooling rate. It should be noted that in industrial manufacturing, NMT products are often exposed to atmosphere for ambient cooling after injection molding. Therefore, we selected three cooling rates to cool NMT products with reference to the cooling rate of the actual manufacturing process: ~1000 °C/min (marked as fast cooling), ~10 °C/min (marked as ambient cooling) and ~1 °C/min (marked as slow cooling).

**Figure 8:**
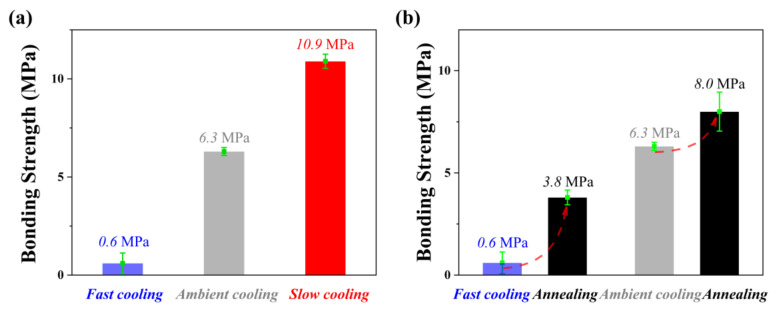
The thermal treatment influence on the bonding strength of PBMA/Al. (**a**) Different cooling rates; (**b**) annealing at 80 °C for 2 h.

As shown in Figure 8a, with the decrease of the cooling rate, the bonding strength was enhanced significantly. Compared with the bonding strength of PBMA/Al after ambient cooling, the bonding strength reduced by 73% after fast cooling, while it increased by 90% after slow cooling. For such unusable NMT products, the bonding strength can be partially maintained after annealing. Both samples show the improvement of bonding strength after annealing at 80 °C for 2 h, as shown in Figure 8b. Especially for fast cooling samples, the bonding strength was increased by more than five-fold after annealing, which is of great significance to improving the eligibility rate of products. It can be expected that the anchor effect of the NMT product can be improved by slow cooling or annealing.

**Figure 9:**
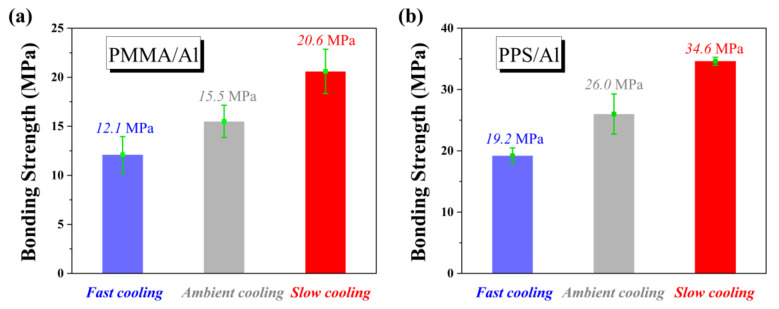
The cooling rate influence on the bonding strength of (**a**) PMMA/Al; (**b**) PPS/Al.

In order to confirm the generality of the above rule, we studied the relationship between the bonding strength and the cooling rate of NMT products consisting of two different polymers and an aluminum substrate. First of all, we selected PMMA, which is similar to PBMA in molecular structure. As shown in Figure 9a, it can be found that the rule is fully consistent with that of PBMA/Al. Then we selected PPS, which is quite different in molecular structure from PBMA. PPS/Al is one of the most widely used NMT products. Figure 9b shows the same rule as that from the above two NMT products. Compared with the bonding strength of PPS/Al after ambient cooling, the bonding strength increased by 34% after slow cooling. This means the bonding strength of NMT products which are already industrially produced can also be improved by slow cooling to meet the increasing requirements.

For a specific polymer, cooling rate is an important factor affecting the interfacial interaction of polymer/metal integrated material, while for different polymers after the same thermal treatment, their physical and chemical properties are more important for the improvement of interfacial interaction. Compared with the bonding strength of three kinds of NMT products after the same thermal treatment, we found the following order: PPS/Al > PMMA/Al > PBMA/Al, which is consistent with the polarity of polymers. The relation between the polymers’ polarity and NMT products bonding strength has also been reported in previous simulation studies [47]. It is reasonable because the interfacial interaction between polar polymer and alumina is stronger than that between nonpolar polymer and alumina. It is worth trying to enhance the bonding strength of NMT products by increasing the polarity of the polymers.

## 4. Conclusions

In this work, we demonstrate that the anchor effect of NMT products is derived from the interaction between the polymer nanorods and nanopore walls at the microscopic level, and such interfacial interaction can be altered by thermal treatments. During the cooling process, the CTE mismatch-induced thermal stress can be controlled by varying thermal treatments. Thermal stress can significantly alter the dynamic behaviors of the polymer chains near the pore walls. After slow cooling or annealing, the polymer nanorods form a heterogeneous glass with a dense interfacial layer in close contact with the pore walls, leading to stable and strong interfacial interaction between the nanorods and pore walls, and then the bonding strength of NMT products is significantly enhanced. Therefore, it is concluded that the treatment which is helpful for polymer nanorods to form a heterogeneous glass will enhance the anchor effect of NMT products. Our work not only provides a facile way to enhance the bonding strength of NMT products, but also deepens the understanding of anchor effect which is important for the development of nanotechnology.

## Data Availability

The data presented in this study are available on request from the corresponding author.

## Supplementary Materials

The following supporting information can be downloaded at: https://www.mdpi.com/article/10.3390/polym14091652/s1, Figure S1: (a) The SEM image of polished aluminum sheet; (b) the SEM image of the aluminum sheet after surface modification, which shows approximately regular pores with a diameter of about 200 nm; (c) the photograph of polished aluminum sheet; (d) the photograph of the aluminum sheet after surface modification.

## References

[1] Brauer M., Wiessner S., Edelmann M., Gedan-Smolka M., Hupfer B., Lehmann D., Nagel J., Schneider K. (2010). Injection Moulding of Metal Plastic Hybrids. Kgk-Kautsch. Gummi Kunstst..

[2] Endemann U., Glaser S., Volker M. (2002). Strong Joint between Plastic and Metal—Assembly Technology for Plastic-Metal Hybrid Structures. Kunstst.-Plast Eur..

[3] Honkanen M., Hoikkanen M., Vippola M., Vuorinen J., Lepisto T. (2009). Metal-Plastic Adhesion in Injection-Molded Hybrids. J. Adhes. Sci. Technol..

[4] Butt J., Shirvani H. (2018). Experimental Analysis of Metal/Plastic Composites Made by A New Hybrid Method. Addit. Manuf..

[5] Taki K., Nakamura S., Takayama T., Nemoto A., Ito H. (2016). Direct Joining of A Laser-Ablated Metal Surface and Polymers by Precise Injection Molding. Microsyst. Technol..

[6] Fabrin P.A., Hoikkanen M.E., Vuorinen J.E. (2007). Adhesion of Thermoplastic Elastomer on Surface Treated Aluminum by Injection Molding. Polym. Eng. Sci..

[7] Roesner A., Scheik S., Olowinsky A., Gillner A., Reisgen U., Schleser M. (2011). Laser Assisted Joining of Plastic Metal Hybrids. Phys. Procedia.

[8] Kimura F., Kadoya S., Kajihara Y. (2016). Effects of Molding Conditions on Injection Molded Direct Joining Using A Metal with Nano-Structured Surface. Precis. Eng..

[9] Lucchetta G., Marinello F., Bariani P.F. (2011). Aluminum Sheet Surface Roughness Correlation with Adhesion in Polymer Metal Hybrid Overmolding. CIRP Ann..

[10] Kim W.S., Yun I.H., Lee J.J., Jung H.T. (2010). Evaluation of Mechanical Interlock Effect on Adhesion Strength of Polymer–Metal Interfaces Using Micro-Patterned Surface Topography. Int. J. Adhes. Adhes..

[11] Masanori N., Naoki A., Masao T., Masao S. (2004). Composite Material of Aluminum Alloy and Resin and Production Method Therefor. U.S. Patent.

[12] Andoh N. (2004). Nano Molding Technology: Aluminum Alloy and Hard Resin Integration Technology by Injection Molding. Seikei-Kakou.

[13] Banea M.D., Rosioara M., Carbas R.J.C., da Silva L.F.M. (2018). Multi-Material Adhesive Joints for Automotive Industry. Compos. Part B Eng..

[14] TaiseiPlas Co. Ltd. (2015). Bonding Technology between Metal and Resin by Injection Molding (NMT) Technical Summary, Product Samples and the Technical Possibilities. J. Soc. Instrum. Control. Eng..

[15] Arai S., Sugawara R., Shimizu M., Inoue J., Horita M., Nagaoka T., Itabashi M. (2020). Excellent Bonding Strength Between Steel and Thermoplastic Resin Using Roughened Electrodeposited Ni/CNT Composite Layer without Adhesives. Mater. Lett..

[16] Horiuti S., Hanada T., Miyamae T., Yamanaka T., Oosumi K., Ando N., Naritomi M. (2012). Analysis of Metal/Plastic Interfaces by Energy-Filtering Transmission Electron Microscopy. J. Adhes. Soc. Jpn..

[17] Sasaki H., Kobayashi I., Sai S., Omoto T., Mori K. (1998). Direct Adhesion of Nylon Resin to Stainless Steel Plates Coated with Triazine Thiol Polymer by Electropolymerization during Injection-Molding. Kobunshi Ronbunshu.

[18] Suzuki N., Kiba S., Kamachi Y., Miyamoto N., Yamauchi Y. (2011). Mesoporous Silica as Smart Inorganic Filler: Preparation of Robust Silicone Rubber with Low Thermal Expansion Property. J. Mater. Chem..

[19] Raghava R.S. (1988). Thermal Expansion of Organic and Inorganic Matrix Composites: A Review of Theoretical and Experimental Studies. Polym. Compos..

[20] Teng C., Li L., Wang Y., Wang R., Chen W., Wang X., Xue G. (2017). How Thermal Stress Alters the Confinement of Polymers Vitrificated in Nanopores. J. Chem. Phys..

[21] Franz C., Lange F., Golitsyn Y., Hartmann-Azanza B., Steinhart M., Krutyeva M., Saalwächter K. (2015). Chain Dynamics and Segmental Orientation in Polymer Melts Confined to Nanochannels. Macromolecules.

[22] Li L., Chen J., Deng W., Zhang C., Sha Y., Cheng Z., Xue G., Zhou D. (2015). Glass Transitions of Poly(methyl methacrylate) Confined in Nanopores: Conversion of Three- And Two-Layer Models. J. Phys. Chem. B.

[23] Li L., Zhou D., Huang D., Xue G. (2013). Double Glass Transition Temperatures of Poly(methyl methacrylate) Confined in Alumina Nanotube Templates. Macromolecules.

[24] Zhang C., Li L., Wang X., Xue G. (2017). Stabilization of Poly(methyl methacrylate) Nanofibers with Core–Shell Structures Confined in AAO Templates by the Balance between Geometric Curvature, Interfacial Interactions, and Cooling Rate. Macromolecules.

[25] Sha Y., Li L., Wang X., Wan Y., Yu J., Xue G., Zhou D. (2014). Growth of Polymer Nanorods with Different Core–Shell Dynamics via Capillary Force in Nanopores. Macromolecules.

[26] Itagaki H., Nishimura Y., Sagisaka E., Grohens Y. (2006). Entanglement of Polymer Chains in Ultrathin Films. Langmuir.

[27] Kadoya S., Kimura F., Kajihara Y. (2019). PBT–Anodized Aluminum Alloy Direct Joining: Characteristic Injection Speed Dependence of Injected Polymer Replicated into Nanostructures. Polym. Test..

[28] Olabisi O., Simha R. (1975). Pressure-Volume-Temperature Studies of Amorphous and Crystallizable Polymers. I. Experimental. Macromolecules.

[29] Rogers S., Mandelkern L. (1957). Glass Transitions of the Poly-(n-Alkyl Methacrylates). J. Phys. Chem..

[30] Shin S., Kim B.S., Kim K.M., Kong B.H., Cho H.K., Cho H.H. (2011). Tuning the Morphology of Copper Nanowires by Controlling the Growth Processes in Electrodeposition. J. Mater. Chem..

[31] Odrobina E., Winnik M.A. (2001). Influence of Entanglements on the Time Dependence of Mixing in Nonradiative Energy Transfer Studies of Polymer Diffusion in Latex Films. Macromolecules.

[32] Schröter K., Reissig S., Hempel E., Beiner M. (2007). From Small Molecules to Polymers: Relaxation Behavior of n-Butyl Methacrylate Based Systems. J. Non-Cryst. Solids.

[33] Suhailath K., Thomas M., Ramesan M. (2021). Studies on Mechanical Properties, Dielectric Behavior and DC Conductivity of Neodymium Oxide/Poly(butyl methacrylate) Nanocomposites. Polym. Polym. Compos..

[34] Morawetz H. (1988). Studies of synthetic-polymers by nonradiative energy-transfer. Science.

[35] Ingratta M., Hollinger J., Duhamel J. (2008). A Case for Using Randomly Labeled Polymers to Study Long-Range Polymer Chain Dynamics by Fluorescence. J. Am. Chem. Soc..

[36] Chan N.Y., Chen M., Dunstan D.E. (2009). Elasticity of Polymer Solutions in Couette Flow Measured by Fluorescence Resonance Energy Transfer (FRET). Eur. Phys. J. E.

[37] Zhou P., Lv P., Yu L., Liu S., Zhang L., Tian C. (2019). Fluorescence Lifetime Based Distance Measurement Illustrates Conformation Changes of Pyl10-CL2 upon ABA Binding in Solution State. Chin. Chem. Lett..

[38] Chan N.Y., Chen M., Hao X.-T., Smith T.A., Dunstan D.E. (2010). Polymer Compression in Shear Flow. J. Phys. Chem. Lett..

[39] Lakowicz J.R. (2006). Principles of Fluorescence Spectroscopy.

[40] Stryer L. (1978). Fluorescence Energy Transfer as A Spectroscopic Ruler. Annu. Rev. Biochem..

[41] Steinberg I.Z. (1971). Long-Range Nonradiative Transfer of Electronic Excitation Energy in Proteins and Polypeptides. Annu. Rev. Biochem..

[42] Qin L., Li L., Sha Y., Wang Z., Zhou D., Chen W., Xue G. (2018). Conformational Transitions of Polymer Chains in Solutions Characterized by Fluorescence Resonance Energy Transfer. Polymers.

[43] Zuo B., Li C., Xu Q., Randazzo K., Jiang N., Wang X., Priestley R.D. (2021). Ultrastable Glassy Polymer Films with an Ultradense Brush Morphology. ACS Nano.

[44] Park J.Y., Mckenna G.B. (2000). Size and Confinement Effects on the Glass Transition Behavior of Polystyrene/o-Terphenyl Polymer Solutions. Phys. Rev. B.

[45] Tsagaropoulos G., Eisenburg A. (1995). Direct Observation of Two Glass Transitions in Silica-Filled Polymers. Implications to the Morphology of Random Ionomers. Macromolecules.

[46] Merino E.G., Duarte P., Fonseca I.M., Danede F., Correia N.T. (2013). Detection of Two Glass Transitions on Triton X-100 under Confinement. J. Phys. Chem. C.

[47] Liu D., Zhou F., Li H., Xin Y., Yi Z. (2020). Study on the Interfacial Interactions and Adhesion Behaviors of Various Polymer-Metal Interfaces in Nano Molding. Polym. Eng. Sci..

