# Supplementary material for: The Influence of Thermal Treatments on Anchor Effect in NMT Products
Source: Polymers (Basel). 2022 Apr 20;14(9):1652. doi: 10.3390/polym14091652 (PMC9104511; doi:10.3390/polym14091652)
Supplement: Supplementary file 1 [file polymers-14-01652-s001.zip › polymers-1671461-supplementary.pdf]

polymers

Supplementary Information

The Influence of Thermal Treatments on Anchor Effect in NMT Products

Huazheng Li [1], Linling Li [2], Ye Sha [1,3], Yuyuan Lu [4], Chao Teng [2], Dongshan Zhou [1], Wei Chen [1,*] and Gi Xue [1]

[1] Department of Polymer Science and Engineering, State Key Laboratory of Coordination Chemistry, Key Laboratory of High Performance Polymer Materials and Technology of Ministry of Education, Nanjing University, Nanjing 210023, China; mg1924041@smail.edu.cn (H.L.); shaye@njfu.edu.cn (Y.S.); dzhou@nju.edu.cn (D.Z.); xuegi@nju.edu.cn (G.X.)
[2] Institute of Critical Materials for Integrated Circuit, Shenzhen Polytechnic, Shenzhen 518055, China; LinlingLi@szpt.edu.cn (L.L.); tengchao@szpt.edu.cn (C.T.)
[3] Department of Chemistry and Material Science, Nanjing Forestry University, Nanjing 210037, China
[4] State Key Laboratory of Polymer Physics and Chemistry, Changchun Institute of Applied Chemistry, Chinese Academy of Sciences, Changchun 130022, China; yylu@ciac.ac.cn
* Correspondence: weichen@nju.edu.cn

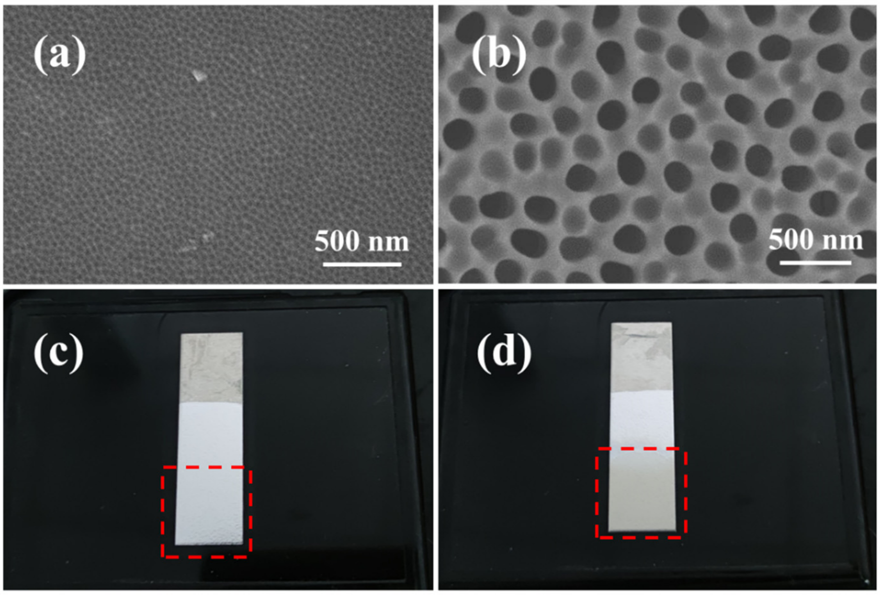

Figure S1. (**a**) The SEM image of polished aluminum sheet; (**b**) the SEM image of the aluminum sheet after surface modification, which shows approximately regular pores with a diameter of about 200 nm; (**c**) the photograph of polished aluminum sheet; (**d**) the photograph of the aluminum sheet after surface modification.
